# Observed effects of “distributional learning” may not relate to the number of peaks. A test of “dispersion” as a confounding factor

**DOI:** 10.3389/fpsyg.2015.01341

**Published:** 2015-09-15

**Authors:** Karin Wanrooij, Paul Boersma, Titia Benders

**Affiliations:** ^1^Amsterdam Center for Language and Communication, University of AmsterdamAmsterdam, Netherlands; ^2^Center for Language Studies, Radboud University NijmegenNijmegen, Netherlands; ^3^School of Psychology, University of NewcastleNSW, Australia

**Keywords:** distributional learning, speech sound acquisition, L2 acquisition, speech perception, confounds in training distributions, measures of dispersion, Bayes factors

## Abstract

Distributional learning of speech sounds is learning from simply being exposed to frequency distributions of speech sounds in one’s surroundings. In laboratory settings, the mechanism has been reported to be discernible already after a few minutes of exposure, in both infants and adults. These “effects of distributional training” have traditionally been attributed to the difference in the *number of peaks* between the experimental distribution (two peaks) and the control distribution (one or zero peaks). However, none of the earlier studies fully excluded a possibly confounding effect of the *dispersion* in the distributions. Additionally, some studies with a non-speech control condition did not control for a possible difference between *processing speech and non-speech*. The current study presents an experiment that corrects both imperfections. Spanish listeners were exposed to either a bimodal distribution encompassing the Dutch contrast /ɑ/∼/a/ or a unimodal distribution with the same dispersion. Before and after training, their accuracy of categorization of [ɑ]- and [a]-tokens was measured. A traditionally calculated *p*-value showed no significant difference in categorization improvement between bimodally and unimodally trained participants. Because of this null result, a Bayesian method was used to assess the odds in favor of the null hypothesis. Four different Bayes factors, each calculated on a different belief in the truth value of previously found effect sizes, indicated the absence of a difference between bimodally and unimodally trained participants. The implication is that “effects of distributional training” observed in the lab are not induced by the number of peaks in the distributions.

## Introduction

### Distributional Learning

The term “distributional learning” refers to learning from simply being exposed to frequency distributions of stimuli in one’s surroundings (Lacerda, 1995; Guenther and Gjaja, 1996). Distributional learning is considered one of the mechanisms with which infants start learning the speech sounds of their native language (e.g., Maye et al., 2002). There is also evidence of this mechanism in adults who try to master difficult non-native speech sound contrasts (e.g., Maye and Gerken, 2000).

**FIGURE 1:**
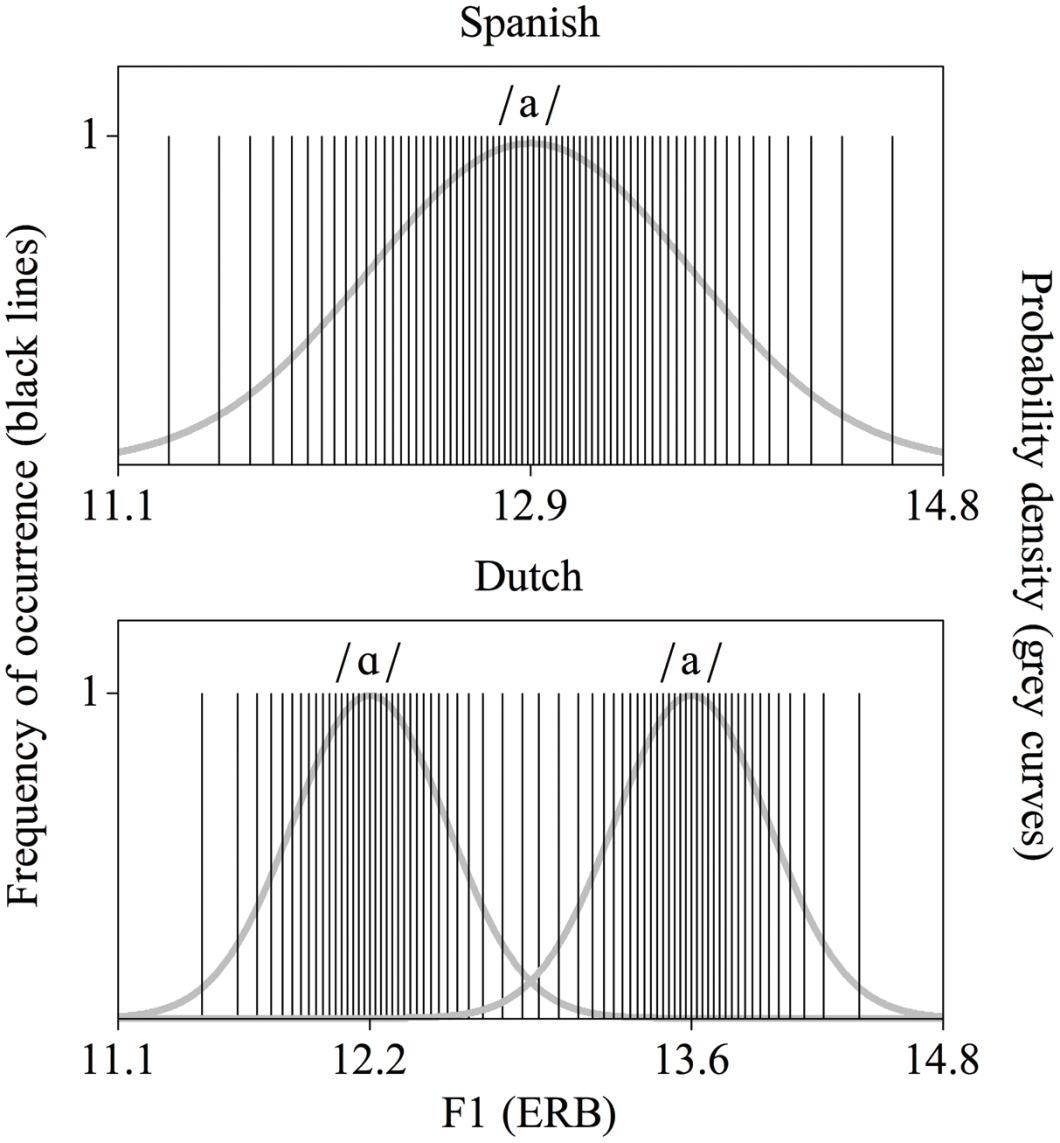
**Distributions of first formant (F1) values (in ERB), representative of the Spanish vowel /a/ **(top)** and the Dutch vowel contrast /ɑ/∼/a/ **(bottom)****. Each solid vertical line represents a hypothetically measured vowel token with a specific F1 value. The grey curves are the underlying probability density functions.

Distributional learning of speech sounds can be explained as follows. When one acoustic property (e.g., the first formant, F1) is measured across many tokens of a certain speech sound category (e.g., a certain vowel), most values are likely to be observed close to the mean of that category. This is illustrated in **Figure 1**. The *x*-axes represent an F1 continuum, for which the F1 values are expressed in ERB (Equivalent Rectangular Bandwidth); each vertical line marks the F1 value hypothetically measured in a token of the Spanish vowel /a/ (**Figure 1**, top), and in a token of the Dutch vowels /ɑ/ or /a/ (**Figure 1**, bottom). It is apparent that the F1 values tend to cluster around certain values, which are the means of the categories. Accordingly, the probability density functions (the grey curves in **Figure 1**) of the F1 values have peaks here. Conversely, the number of peaks observed in a probability density function is indicative of the number of speech sound categories along the corresponding acoustic continuum. Frequency distributions such as the schematic one in **Figure 1** have been observed for several speech sound categories (e.g., Lisker and Abramson, 1964; Newman et al., 2001; Lotto et al., 2004).

Distributional learning implies that exposure to such speech sound distributions induces listeners to perceive tokens with acoustic values that occur within one peak as exemplars of the same speech sound category. The idea is that exposure to the Dutch language, and thereby to the F1 distribution at the bottom of **Figure 1**, prepares Dutch listeners for perceiving vowel tokens with F1 values of around 12.2 ERB as belonging to one speech sound category (namely /ɑ/), and vowel tokens with F1 values of around 13.6 ERB as belonging to another speech sound category (namely /a/), while exposure to the Spanish language, and thereby to the F1 distribution at the top of **Figure 1**, prompts Spanish listeners to perceive these same vowel tokens as exemplars of one single speech sound category (namely Spanish /a/).

The just-described distributional-learning mechanism has been tested empirically in the lab, where perceptual tuning to the number of peaks in the input distribution has been reported to occur already after a few minutes of exposure, for both infants and adults (for infants: Maye et al., 2002, 2008; Yoshida et al., 2010; Capel et al., 2011; Wanrooij et al., 2014; for adults: Maye and Gerken, 2000, 2001; Shea and Curtin, 2006; Gulian et al., 2007; Hayes-Harb, 2007; Escudero et al., 2011; Wanrooij and Boersma, 2013; Wanrooij et al., 2013; Escudero and Williams, 2014). In a typical distributional-learning experiment, two groups of participants (e.g., native speakers of Spanish) are exposed to speech sound distributions encompassing a not yet acquired speech sound contrast (e.g., the Dutch vowel contrast /ɑ/∼/a/): one group is presented with a *unimodal* training distribution (i.e., with *one peak*, as in an F1 distribution of the Spanish vowel /a/) and another group with a *bimodal* training distribution (i.e., with *two peaks*, as in an F1 distribution of the Dutch vowel contrast /ɑ/∼/a/). Such training distributions have been “discontinuous” or “continuous” (Wanrooij and Boersma, 2013). Discontinuous distributions contain only a limited number of acoustically different stimuli, which are each repeated a certain number of times according to the respective distribution. (Examples of discontinuous distributions are shown in **Figure 3** in “No Adequate Control for Dispersion Across Distributional Learning Studies”). Continuous distributions consist of a large number of acoustically different stimuli, each of which is presented only once. The acoustic values are chosen to be such that they match the intended probability density function. (Examples of continuous distributions are shown in **Figure 4** in “Training”). After exposure to the speech sound distribution, participants are tested on their discrimination or categorization of representative tokens of the contrast involved (e.g., [ɑ]- and [a]-tokens). If the distributional-learning mechanism is effective, it is expected that bimodally trained participants will discriminate or categorize these test stimuli better than unimodally trained participants. This difference between the groups is expected because only the bimodally trained participants have been exposed to a distribution that suggests the existence of a contrast between the two categories.

### Problems in Previous Research on Distributional Learning

Studies on distributional learning (previous section) have focused on the *number of peaks* as the relevant factor that shapes the distributional learning process. Unfortunately, it is not certain that the reported effects of distributional learning in these studies were truly due to perceptual changes induced by the number of peaks in the distributions. The chosen methodologies leave open the possibility that other factors caused these reported effects. Specifically, none of the earlier studies fully equated the training distributions on the amount of *dispersion*, as expressed in for instance the range and the standard deviation (SD) of the acoustic values (see “No Adequate Control for Dispersion Across Distributional Learning Studies”). The lack of control for dispersion may be an important oversight in the light of indications that the dispersion of acoustic values in the training stimuli can affect speech sound acquisition (see “The Role of Dispersion in Speech Sound Learning”). Evidence even exists that measures of dispersion (such as the range and the SD) in a training distribution may exert more influence on perception than measures of central tendency (such as the mean; Holt and Lotto, 2006, p. 3066). A second possible confounding effect in some studies with a non-speech control group, is the effect of *processing speech versus non-speech* (see “No Adequate Control for Processing Speech versus Non-Speech”). The two potential confounding factors are discussed in turn.

### The Role of Dispersion in Speech Sound Learning

**FIGURE 2:**
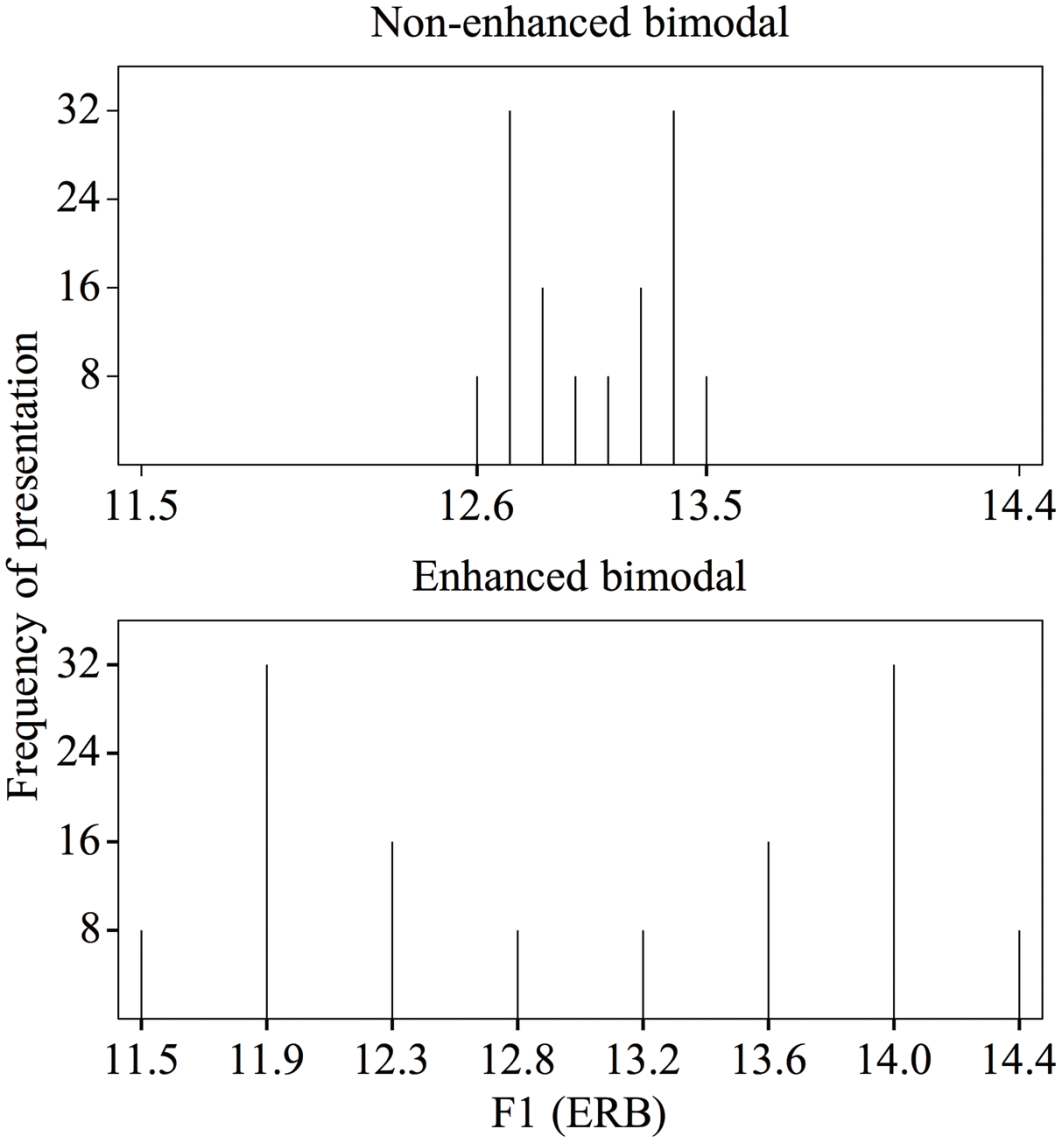
**Non-enhanced **(top)** and enhanced **(bottom)** bimodal distributions of F1 values in the Dutch vowel contrast /ɑ/∼/a/, as used in Escudero et al. (2011) and Wanrooij et al. (2013)**.

Indications that the dispersion of the acoustic values in speech sound distributions can influence adults’ speech sound learning can be found in studies reporting that training with “enhancement” leads to changes in adults’ perception (e.g., Jamieson and Morosan, 1986). Enhancement refers to the widening of the acoustic distance between speech sound categories, thereby affecting the dispersion in the presented stimulus distributions. The precise effect of enhancement on the dispersion depends on the way in which it is implemented in the training paradigm. In distributional training experiments, it has been implemented by giving enhanced bimodal distributions a larger acoustic difference between the means (i.e., the two peaks in the distribution^1^, each of which represents a speech sound category), a wider range, and a larger SD than non-enhanced bimodal distributions (Escudero et al., 2011; Wanrooij et al., 2013)^2^. These three factors are of course strongly interdependent. **Figure 2** demonstrates the difference between the non-enhanced (top) and enhanced (bottom) distributions.

In other training experiments, where participants typically receive feedback during categorization training, enhancement has been implemented by “perceptual fading” (Jamieson and Morosan, 1986), a technique originally applied to visual discrimination learning in birds (Terrace, 1963). With this technique, participants are first presented with exemplars of each speech sound category whose acoustic properties are “enhanced,” thus presumably making it easier to hear a difference between the categories. If the participant categorizes the exemplars well, the acoustic difference between the categories is reduced in small steps. As the actually presented distributions depend on participants’ performance and thus vary per participant, studies using this technique do not always specify the distribution in terms of means and measures of dispersion. Nevertheless, the initial enhancement is likely to widen the dispersion of the presented distributions in comparison to distributions without such enhancement.

Although direct comparisons between the effects of enhanced and non-enhanced training tend to yield non-significant results (e.g., Iverson et al., 2005; Escudero et al., 2011), enhanced training (both enhanced distributional training and training with perceptual fading) generally leads to improved categorization or discrimination of the trained speech sound categories after as compared to before training (Jamieson and Morosan, 1986; Iverson et al., 2005; Kondaurova and Francis, 2010) and in addition sometimes also as compared to a control group that received no training with speech sound stimuli (McCandliss et al., 2002; Escudero et al., 2011; Wanrooij and Boersma, 2013; Wanrooij et al., 2013). These improvements leave open the possibility that enhancement of the speech sounds presented during training (likely affecting the range and the SD of a speech sound distribution) indeed affects speech sound learning in adults.

The observed benefit of enhancement in distributional training studies could be due to better distributional learning (Escudero et al., 2011; Wanrooij et al., 2013). However, the assumed benefit of enhancement in perceptual fading studies is usually not attributed to better distributional learning but to a facilitation of “attentional learning,” i.e., learning through focusing one’s “attention” on the relevant differences between speech sound categories (e.g., Jamieson and Morosan, 1986; Francis and Nusbaum, 2002; Iverson et al., 2005; Kondaurova and Francis, 2010). Such attentional learning is also raised as an additional explanation (apart from better distributional learning) for improved categorization after training in distributional training studies (Escudero et al., 2011; Wanrooij et al., 2013; Escudero and Williams, 2014). Perceptual fading studies that focus on attentional learning generally leave the concept of attention undefined, but it looks as if attention in these studies is mediated by existing knowledge (about, for instance, native speech sound categories; Logan et al., 1991, p. 882) or knowledge obtained during the experiment in the form of feedback (e.g., McCandliss et al., 2002). Such attention can be related to top–down processes in the brain (Posner and Petersen, 1990; Roelfsema, 2011). Attentional learning thus seems to contrast with distributional learning, which is viewed as a purely stimulus-driven, bottom-up process (Lacerda, 1995; Guenther and Gjaja, 1996).

At the same time, our understanding of attentional learning and distributional learning (assuming that they exist) is poor, and it is difficult to establish that they are truly separate processes. For instance, *both* predict that the learning of a speech sound contrast should improve from enhancement if enhancement is implemented by only pulling the means of the two categories wider apart without changing each peak’s SD. Such an enhancement method could draw participants’ attention to the differences between the categories (thus advancing attentional learning) *and* would reduce the overlap between the two peaks (thus promoting distributional learning)^3^. Accordingly, improvement of discrimination or categorization performance after such enhanced distributional training could be accounted for by both distributional learning and attentional learning. Experiments designed to demonstrate the existence of the distributional learning mechanism must exclude the possibility that the results can be explained through attentional learning, and must thus use the same dispersion in the experimental (two peaks) and the control (one or zero peaks) distributions.

In sum, even though it is still unclear precisely what role measures of dispersion in distributions play in adults’ speech sound learning, there are several indications that such measures do play a role. Accordingly, it is important to exclude a possibly confounding influence of dispersion in distributional training experiments. An equal dispersion in the distributions to be compared would also reduce the possibility that differences in attentional learning between training conditions could account for the results, rather than differences in distributional learning.

### No Adequate Control for Dispersion Across Distributional Learning Studies

None of the previous studies on distributional learning, neither those with infants nor those with adults (see “Distributional Learning”), fully excluded dispersion as a possible factor that can account for the observed differences between the bimodal training groups and the control groups. Three possible measures of dispersion are the range, the SD, and the “edge strength.” These are discussed here in turn.

**FIGURE 3:**
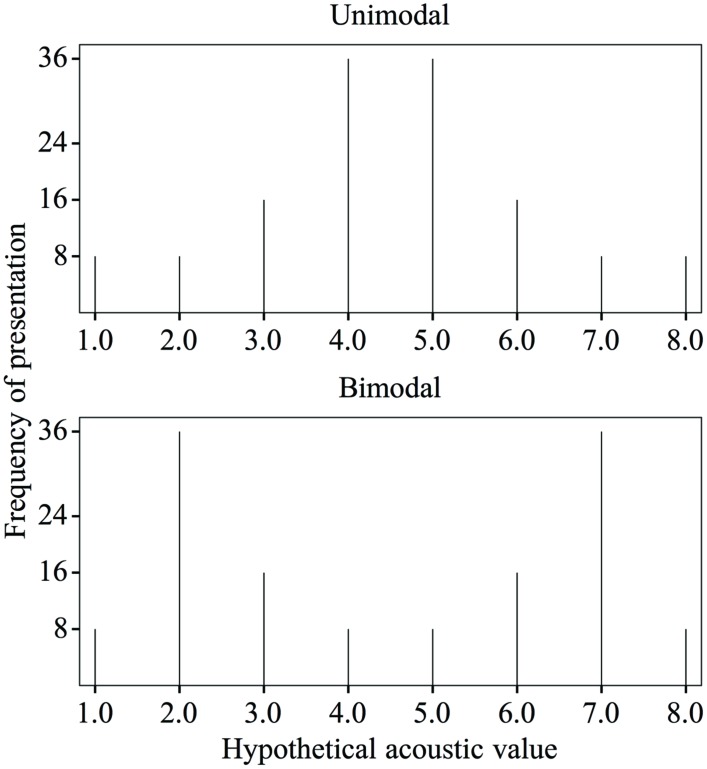
**Unimodal **(top)** and bimodal **(bottom)** training distributions of a hypothetical acoustic value (with an equal psychoacoustic distance of 1 between subsequent values along the continuum), with the frequencies of presentation as used in Maye et al. (2008, p. 125)**.

The first measure of dispersion is the range. Typical bimodal and unimodal distributions such as those in Maye et al. (2008) have the same range within a study: the minimum and maximum presented values are the same in the one as in the other distribution (see **Figure 3**). Range was not excluded as a possibly confounding effect in four studies on distributional learning that used a music control group instead of a unimodal control group (Escudero et al., 2011; Wanrooij and Boersma, 2013; Wanrooij et al., 2013; Escudero and Williams, 2014). These four studies investigated the effect of distributional training on Spanish listeners’ categorization of vowel tokens representing the Dutch vowel contrast /ɑ/∼/a/. In all four studies, listeners to an enhanced bimodal distribution improved significantly more in categorization accuracy than listeners to music^4^. This result could be due to distributional learning, and thus to the presence of two peaks in the enhanced bimodal distribution. However, the use of a music control group instead of a unimodal control group leaves open the possibility that the reported effect is related to the wide range of presented acoustic values in the enhanced bimodal distribution.

The second measure of dispersion, the SD, is larger for the bimodal distribution than for the unimodal distribution across studies with a unimodal control group. For instance, if we take typical unimodal and bimodal distributions with stimulus frequencies as in Maye et al. (2008) and if we take a hypothetical acoustic continuum in which each step along the continuum has an identical psychoacoustic distance of 1 (see **Figure 3**), the SD of the unimodal distribution is 1.7 and that of the bimodal distribution is 2.3^5^. In studies with a music control group, the SD of the (enhanced) bimodal distribution cannot be compared to that of the music condition, so that here too (i.e., just as in the studies with a unimodal control group) the possibility remains open that the reported effects of distributional training are related to the large SD in the bimodal distribution rather than to the presence of two peaks.

Our third measure of dispersion is the “edge strength.” This term refers to the density of stimuli in the leftmost and rightmost tails of the distribution (the “edges”). It is conceivable that a large edge strength can draw participants’ attention to the relevant differences between stimuli, just as a wide range and SD may do (see “The Role of Dispersion in Speech Sound Learning”). Specifically, the more stimuli are sampled at the edges rather than in the middle of the distribution, the more the listeners’ attention can be drawn toward the end points of the continuum, rather than toward the middle. In view of the above, the reported effect of distributional training in the studies with a music control group may have been due to the large edge strength in the enhanced bimodal distribution rather than to the presence of two peaks. Many studies with a *unimodal* control group and an eight-step discontinuous distribution ensured that the stimuli with minimum and maximum values were equally frequent in the unimodal and the bimodal training (e.g., Maye et al., 2008; see **Figure 3**: stimuli number 1 and 8 were each presented eight times in both distributions). Thus, when computed with edges at 1/8 of the range, the bimodal and unimodal distributions in these studies have equal edge strengths. However, when computed with edges at a larger portion (e.g., 1/6) of the range, the bimodal distributions have greater edge strength. This illustrates that the edge strength depends on the chosen width of the edges. Since it is not known how wide edges must be to avoid a confounding influence of attention to the edges, it remains a possibility that the reported effect of distributional training in the studies with a unimodal control group (just as in the studies with a music control group) was based on a larger edge strength in the bimodal group than in the control group.

In sum, previous research on distributional learning has not fully excluded a possible learning effect based on measures of dispersion, such as the range (in some studies), the SD (in all studies), and the edge strength (depending on the choice of the edges in some or all studies).

### No Adequate Control for Processing Speech versus Non-Speech

A significant difference in categorization improvement after distributional training between a group exposed to an enhanced bimodal distribution and a group exposed to music (Escudero et al., 2011; Wanrooij and Boersma, 2013; Wanrooij et al., 2013; as discussed in “No Adequate Control for Dispersion Across Distributional Learning Studies”) could not only be attributed to a difference in the number of peaks or to a difference in the dispersion of the acoustic values between the two conditions (as explained in “No Adequate Control for Dispersion Across Distributional Learning Studies”), but also more generally to a difference between *processing speech* as during the enhanced bimodal training and *processing non-speech* as during the musical training phase. Differences in processing speech versus non-speech are well-documented and include indications that speech is processed along different routes in the brain than non-speech (e.g., Dehaene-Lambertz et al., 2005). Such differences are not related to distributional learning, which is supposedly not based on different processing routes during the bimodal training than the control training, but rather, as supported by computer simulations, on a different tuning of neurons in low-level cortical areas such as the primary auditory cortex (Guenther and Gjaja, 1996).

In sum, the previously reported effects of distributional training in studies with only a non-speech control group could be related to a difference between processing speech and processing non-speech rather than to a difference in the number of peaks in the distribution.

### Solving the Problems: an Equally Wide Unimodal Control Distribution

The present study followed four previous distributional training studies (Escudero et al., 2011; Wanrooij and Boersma, 2013; Wanrooij et al., 2013; Escudero and Williams, 2014) in the choice of the population and of the vowel continuum appropriate for these listeners: native speakers of Spanish were exposed to distributions along the spectral contrast between the Dutch vowels /ɑ/ and /a/. /a/ has a higher F1 and a higher second formant, F2 (Pols et al., 1973; Adank et al., 2004). This spectral contrast is difficult to learn to perceive for Spanish listeners (Escudero et al., 2009; Escudero and Wanrooij, 2010), but it is the main cue for most native speakers of Dutch (Van Heuven et al., 1986; Escudero et al., 2009). Also in line with the four previous studies, participants were tested on their categorization accuracy of naturally produced [ɑ]s and [a]s before and after training.

In order to determine whether the *number of peaks* (factor 1) in a speech sound distribution tunes participants’ perception, and is thus the factor behind the results in distributional-learning experiments, it was necessary to exclude *dispersion* (factor 2) and *processing differences between speech and non-speech* (factor 3) as possible confounding factors. This can be done by using an experimental distribution and a control distribution that only differ in the number of peaks (factor 1 still present), and which thus have an equal dispersion (factor 2 excluded) and are both speech sound distributions (factor 3 excluded).

The experimental distribution in the current study was based on the “enhanced” bimodal distribution used by Escudero et al. (2011) and Wanrooij et al. (2013) for the same continuum and population, because these studies found a significantly better improvement in vowel categorization after exposure to this distribution than after exposure to music. The control distribution in the present study was a unimodal distribution of speech sounds with the same dispersion (as defined by the range, SD and edge strength; see “No Adequate Control for Dispersion Across Distributional Learning Studies”) as this bimodal distribution. We will henceforth refer to the participants listening to the bimodal distribution as the *Bimodal* group, and to the participants presented with the unimodal distribution as the *Unimodal* group.

By using bimodal and unimodal distributions with an equal dispersion, we rule out the possibility that differences in improvement of categorization between the Bimodal and Unimodal groups can be due to differences in dispersion (factor 2). By using only speech sound distributions, we preclude that dissimilar processing of speech versus non-speech (factor 3) plays a role in any differences found between the two groups. Thus, if we find that the Bimodal group improves significantly more than the Unimodal group, we can confidently attribute this difference to an effect of the number of peaks (factor 1). There will be no straightforward explanation if the reverse result occurs, i.e., if the Unimodal group improves more than the Bimodal group.

If no significant difference (in terms of *p*-values) between the two groups emerges, we are confronted with a *null result* that does not allow us to conclude whether the number of peaks plays a role or not. This problem will be addressed by the computation of Bayes factors (e.g., Kass and Raftery, 1995; Rouder et al., 2009), which allow us to quantify the relative credibilities of the alternative hypothesis (e.g., that the Bimodal group will improve by a certain amount more than the Unimodal group) *and* the null hypothesis (that there will not be a difference in improvement between the two groups).

## Materials and Methods

Unless stated otherwise, the method was identical to that used in Escudero et al., 2011 (henceforth: EBW2011), Wanrooij et al., 2013 (henceforth: WER2013) and Wanrooij and Boersma, 2013 (henceforth: WB2013). Spanish adult learners of Dutch (see “Participants”) went through a training phase (see “Training”), and before and after this training they performed a test that assessed their categorization of several Dutch [ɑ]- and [a]-tokens (see “Pre- and Post-Tests”). A comparison of post-test to pre-test accuracy scores determined participants’ improvement in categorization performance.

### Participants

The participants were adult native speakers of Spanish, who had been raised monolingually, at least until the age of 18. They were semi-randomly assigned to either the Unimodal group or to the Bimodal group (see “Solving the Problems: an Equally Wide Unimodal Control Distribution”), each eventually containing 60 participants. Assignment to the groups was not completely random, because we balanced the groups in terms of age, sex and length of residence in the Netherlands, in this order of importance. **Table 1** presents the mean age, age range and mean length of residence, in the Unimodal (32 men, 28 women) and Bimodal (26 men, 34 women) groups.

**Table 1:** Participants’ age, age range, and length of residence (in years) in the Netherlands, and Dialang score, for the Unimodal and Bimodal groups.

Group	Mean age	Age range	Mean length of residence	Dialang score
Unimodal	30.2 (7.3)	20.0–56.3	1.2 (1.4)	2.27 (1.28)
Bimodal	31.0 (8.0)	18.7–52.6	1.4 (2.0)	2.25 (1.42)

*The numbers between parentheses give the *SDs* within each group*.

Previous research has shown that experience with new languages after adolescence does not significantly alter the perception of isolated vowels (e.g., Dutch adults listening to English vowels: Schouten, 1975; Broersma, 2005; Catalan adults listening to English vowels: Cebrian, 2006; Spanish adults listening to Dutch vowels: Escudero and Wanrooij, 2010). Therefore, we did not expect such experience to affect our results. Nevertheless, we examined whether there was a difference between the Unimodal and Bimodal groups in the participants’ second language profiles. Such differences were not observed. Nearly all participants had experience with English (57 in Unimodal, 59 in Bimodal). Many indicated to have experience with Dutch (17 in Unimodal, 23 in Bimodal) or another language (23 in Unimodal, 22 in Bimodal). To pinpoint the level of Dutch, participants did a Dialang general listening comprehension test (http://www.lancaster.ac.uk/researchenterprise/dialang/about; Alderson and Huhta, 2005) after the distributional training experiment, just as in EBW2011 and WER2013. **Table 1** lists the mean Dialang scores per group (Dialang has six levels: A1, A2, B1, B2, C1, and C2, which we converted to scores running from 1 to 6. Hence, the lowest possible mean score is 1 and the highest is 6). Just as in EBW2011 and WER2013, there was no significant difference in the Dialang scores between the Unimodal and Bimodal participants (Mann–Whitney *U*-test, *p* = 0.55).

This study was carried out in accordance with the recommendations of the Ethical Committee of the Faculty of Humanities of the University of Amsterdam. All participants signed informed consent forms.

### Stimuli and Procedure

#### Training

**FIGURE 4:**
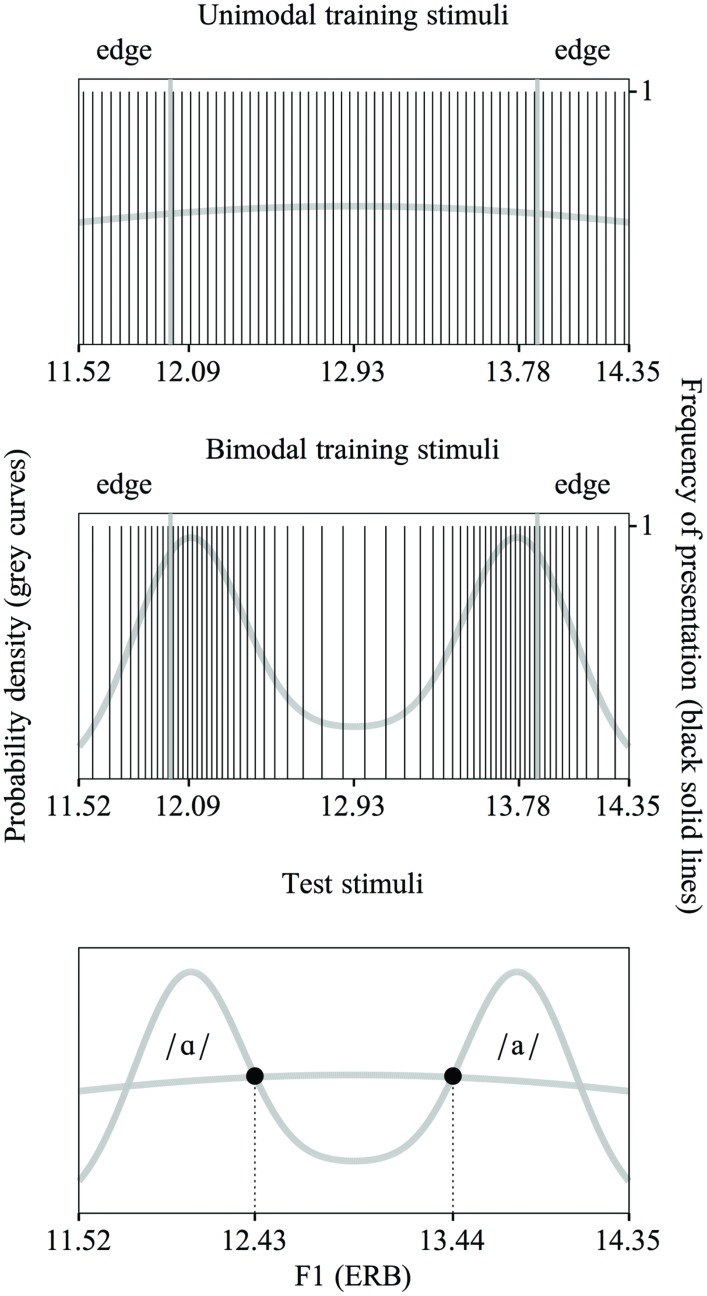
**The unimodal **(top)** and bimodal **(middle)** training distributions of F1 values used in the present experiment, with an equal range and a nearly equal SD and edge strength (explanation: see text)**. The unimodal distribution represents the Spanish vowel /a/ and the bimodal distribution is representative of the Dutch vowel contrast /ɑ/∼/a/. Each vertical line shows the F1 value of a single stimulus. (For the purpose of clarity only 64 values are shown, rather than the 256 values used). Test stimuli in the present experiment **(bottom)**. The F1 values of the test stimuli lie at the intersections of the two distributions.

**Figure 4** shows the unimodal (top) and bimodal (middle) training distributions used in the current experiment. The unimodal distribution is representative of the Spanish vowel /a/ and the bimodal distribution is representative of the Dutch vowel contrast /ɑ/∼/a/. As is apparent in **Figure 4**, we created continuous (see “Distributional Learning”) distributions, just as in WB2013 and in contrast to EBW2011 and WER2013. The training stimuli were made with the Klatt synthesizer in the program Praat (Boersma and Weenink, 2013) in line with the procedure described in WB2013. The manipulated acoustic dimensions were F1 and F2. Only the F1 continuum is shown in **Figure 4**.

Just as in WB2013, the bimodal distribution was created on the basis of two Gaussian curves. The means and SDs were slightly adapted from the previously used values (see below) to accommodate the requirement that both distributions should have the same dispersion (see “Solving the Problems: an Equally Wide Unimodal Control Distribution”). The unimodal distribution was created on the basis of a single Gaussian curve.

We defined the dispersion of the distributions with the three variables that were also mentioned in the Introduction (see “No Adequate Control for Dispersion Across Distributional Learning Studies”): the range, the SD and the edge strength. The *range* of both distributions was set to run from 11.52 to 14.35 ERB for F1 (as is visible in **Figure 4**) and from 15.29 to 18.15 ERB for F2. The term “range” below applies to both F1 values and F2 values. We positioned the means of the underlying bimodal Gaussians at 20 and 80% of the range, and set the SD of these underlying Gaussians at 10% of the range. In addition, we skewed the two peaks in the distribution slightly outward^6^. The mean of the underlying unimodal Gaussian was placed at 50% of the range and had a SD of 100% of the range. With these settings, the *SDs* of the bimodal and unimodal training distributions were similar, namely 29.3 and 28.4% of the range respectively^7^. The two edges for determining the *edge strength* were each placed at 1/6 of the range of the distribution (see **Figure 4**). With the settings for the range and the SDs as outlined above (this section), the edge strength was 0.954 for the unimodal distribution and 0.933 for the bimodal distribution. These numbers are based on a normalized distribution, i.e., a distribution with a range from 0 to 1 and a mean probability density of 1. **Table 2** summarizes the ranges of F1 and F2 values, the SDs and edge strengths of the unimodal and bimodal distributions.

**Table 2:** Three measures for the dispersion of the unimodal and bimodal distributions: the range of F1 and F2 values, the SD and the edge strength.

Distribution	Range F1 (ERB)	Range F2 (ERB)	SD (% of range)	Edge strength
Unimodal	11.52–14.35	15.29–18.15	28.4	0.954
Bimodal	11.52–14.35	15.29–18.15	29.3	0.933

It was not simple to obtain a unimodal and bimodal distribution that were as equal as possible in all three measures of dispersion. The chosen *range* was identical to the range of the enhanced bimodal distributions in EBW2011, WER2013 and WB2013. Widening the F1 and F2 range would lead to including vowels extending into the /ɔ/- region, so that the bimodal distribution would be more representative of the /ɔ/∼/a/ contrast than the /ɑ/∼/a/ contrast. Shrinking the F1 and F2 range would make the test stimuli too similar. (In order to ensure the discriminability of the test stimuli, we required them to be at least 1 ERB apart in F1 and F2. As will be explained in “Pre- and Post-Tests,” the acoustic values of the test stimuli were based on the intersections of the training distributions. Shrinking the range would shorten the acoustic distance between the intersections too much).

The *SDs* of the unimodal and bimodal distributions could only be made similar by adapting the distribution in WB2013. That distribution had been created on the basis of the sum of two Gaussians with means at 25 and 75% of the range, and each with a SD of 11% of the range. The SD of the resulting distribution was 26.8% of the range. In order to make the SD of the unimodal distribution similar to this percentage, while at the same time ensuring that (1) the range would remain as determined, (2) the acoustic distance between the test stimuli [ɑ] and [a] would not become too small (as just explained), and (3) the edge strength in 1/6 of the edges remained similar in both distributions, the enhanced bimodal distribution of WB2013 had to be adapted by changing the means and SD of the Gaussians, and introducing some skewness (as specified above).

If distributional learning would occur, a small effect size (i.e., of the difference in categorization improvement between unimodally and bimodally trained participants) could be expected. This is because EBW2011, WER2013, and WB2013 found 95% confidence intervals close to zero when they quantified the difference in improvement in the categorization of Dutch [ɑ]- and [a]-tokens between Spanish listeners exposed to an enhanced bimodal distribution of Dutch /ɑ/∼/a/ and Spanish listeners in the control condition. To increase the chance of detecting such a small effect, we used twice as many stimuli in the training distributions as in these previous studies, namely 256 in each distribution. (For the purpose of clarity, only 64 stimulus values are shown in each distribution in **Figure 4**).

Following several distributional learning studies with a unimodal control group (Maye and Gerken, 2000, 2001; Shea and Curtin, 2006; Hayes-Harb, 2007), we added fillers to the training stimuli. Specifically, the 256 experimental training stimuli were supplemented by 128 fillers, of which 64 were tokens of Dutch [i] and 64 were tokens of Dutch [u]. The F1 values of these fillers were sampled randomly from Gaussian distributions (one for each vowel), with a mean set at 50% of the range and a SD of 30% of the range. The F1 range was 5.81–6.93 ERB for both vowels. The F2 values were generated in the same way. The F2 range was 22.10–23.46 ERB for [i] and 10.84–12.20 ERB for [u]. Just as the stimuli in the training distributions, the fillers were created with the Klatt synthesizer in Praat (Boersma and Weenink, 2013).

Each stimulus presented during the training phase (i.e., each experimental stimulus and each filler) had a fundamental frequency (F0) contour that declined from 150 to 100 Hz and a duration of 140 milliseconds (ms). The durational difference between /ɑ/ and /a/ (/a/ is longer; Adank et al., 2004) did not appear in the training distributions, so that participants could only hear the spectral difference, which is difficult to perceive for these Spanish listeners (Escudero et al., 2009; Escudero and Wanrooij, 2010; see “Solving the Problems: an Equally Wide Unimodal Control Distribution”).

The order of presentation of the 384 stimuli (=256 experimental stimuli + 128 fillers) was randomized for each participant individually. The stimuli were presented with an offset-to-onset inter-stimulus interval (ISI) of 750 ms. The total duration of the training was 5.7 minutes. Participants were asked to listen to the training vowels carefully, because they would perform a post-test afterward.

#### Pre- and Post-Tests

The pre- and post-tests were identical XAB categorization tasks, which were the same as in EBW2011, WER2013, and WB2013 except for the two response options A and B (see below). Each of the 80 trials presented participants with a natural token (the X-stimulus) of [ɑ] or [a], followed by two synthetic response options (the A- and B-stimuli), which were [ɑ] followed by [a] or reverse. There were 40 unique X-stimuli, which were a subset of the corpus reported by Adank et al. (2004). Twenty stimuli were [ɑ] and 20 were [a]. Ten stimuli of each vowel were produced by men and 10 by women. Each X-stimulus appeared twice in each test, once with the response options in the order [ɑ] – [a] and once with the response options in the reverse order.

The response options A and B were created with the Klatt synthesizer in Praat (Boersma and Weenink, 2013). In order to ensure that the F1 and F2 values of these response options were trained equally intensively in the unimodal and bimodal distributions, we calculated the intersections of the two distributions (the circles in **Figure 4**, bottom). These values differed slightly from the ones used in EBW2011, WER2013 and WB2013, namely for [ɑ] F1 = 12.44 ERB, F2 = 16.21 ERB, and for [a] F1 = 13.43 ERB, F2 = 17.23 ERB^8^. Each response option had the same F0 contour (i.e., declining from 150 to 100 Hz) and duration (140 ms) as the training stimuli. The duration was the same for both options in order to isolate participants’ learning of the spectral contrast (see “Training”).

Before the pre-test and the post-test, participants performed a practice test with [i] and [y] stimuli to make sure that they understood the test, and that they did not have problems hearing the vowels^9^.

## Analyses and Results

### Descriptives

**Table 3** lists the pre-test and post-test accuracy percentages, and the difference (i.e., the post-test minus the pre-test accuracy percentage), for the Unimodal and Bimodal groups separately. This difference is a measure of improvement after training, and thus reflects the *improvement score*.

**Table 3:** Pre- and post-test accuracy percentages, and improvement score (=post- minus pre-test accuracy percentage) per group.

Group	Pre	Post	Improvement
Unimodal	60.35 (10.28)	66.33 (12.07)	5.98 (8.32)
Bimodal	59.98 (10.03)	65.25 (13.57)	5.27 (9.62)

*Standard deviations between participants in each group are given between parentheses*.

### Significance Tests

The first set of analyses is based on common (frequentist) significance testing. This was done to assess the outcomes in the context of the previous results on distributional learning in Spanish adults presented with distributions of Dutch /ɑ/∼/a/ (EBW2011, WER2013, WB2013), which were all based on such tests.

In line with EBW2011, WER2013, and WB2013, we performed a one-sample *t*-test for each group (i.e., one for Unimodal and one for Bimodal), that compared the group’s improvement score against zero. The results show a significant difference from zero, and thus better categorization accuracy after than before training, for both groups (Unimodal: 95% confidence interval [henceforth CI] = +3.83 ∼ +8.13%, *t*[59] = 5.56, *p* < 0.0001, standardized effect size *d* = 0.72; Bimodal: CI = +2.79 ∼ +7.76%, *t*[59] = 4.25, *p* < 0.0001, *d* = 0.55^10^). Accordingly, both unimodal and bimodal training yield improved categorization performance for Spanish learners of Dutch /ɑ/∼/a/.

An independent-samples (Unimodal versus Bimodal) *t*-test, with the improvement score as the dependent variable, did *not* show a significant difference between the Unimodal and Bimodal groups (mean difference in improvement score, i.e., Bimodal – Unimodal score = –0.71%, CI = –3.96 ∼ +2.54%, *t*[118] = –0.43, *p* = 0.67, *d* = –0.08^11^). This result does not enable us to say with confidence that Spanish learners’ perception of Dutch /ɑ/∼/a/ is affected by the number of peaks in a training distribution.

### Bayes Factors

From having found a *p*-value above 0.05 we cannot draw any conclusions about whether the null hypothesis is true or false. Because we wanted to be able to quantify evidence in favor of both the alternative *and* the null hypothesis, we computed Bayes factors (henceforth “BFs”) (e.g., Kass and Raftery, 1995; Gallistel, 2009; Rouder et al., 2009; Kruschke, 2010). A BF denotes the likelihood ratio of the data occurring under the null hypothesis (*H_0_*) versus the data occurring under the alternative hypothesis (*H_1_*):

$${BF}_{01}=\frac{p(data\,\;\,|H_{0})}{p(data\,\;|H_{1})}$$

The “01” in this equation refers to *H_0_* and *H_1_* respectively. Thus, if BF_01_ = 10, the observed data are 10 times more likely to occur if *H_0_* is true than if *H_1_* is true; if BF01 = 0.1, the observed data are 10 times more likely to occur if *H_1_* is true than if *H_0_* is true. If we assume that *H_0_* and *H_1_* are equally likely *a priori* (as is common and as we do henceforth), the Bayes factor BF_01_ can be said to quantify the evidence in support of *H_0_* over *H_1_*. Thus, if BF_01_ = 10, *H_0_* is 10 times more likely to be true than *H_1_* (i.e., the odds are 10 to 1 in favor of *H_0_*); if BF_01_ = 0.1, *H_1_* is 10 times more likely to be true than *H_0_* (i.e., the odds are 10 to 1 in favor of *H_1_*). Whether a clear choice between the two hypotheses is possible, depends on the magnitude of the Bayes factor. If BF_01_ > 20, there is said to be strong support for *H_0_*, and if BF_01_ < 1/20, there is said to be strong support for *H_1_*; if, however, BF_01_ lies between 3 and 20, the data are said to moderately favor *H_0_*, and if BF_01_ lies between 1 and 3, the data are said to only trivially favor *H_0_* (Kass and Raftery, 1995).

In the current paper, the null and alternative hypotheses are defined in terms of the standardized effect size of the difference in the improvement score (=the post-test minus the pre-test accuracy percentage) between the Unimodal and Bimodal groups, i.e., in terms of how much the two groups differ in their improvement of categorization accuracy after as compared to before training. An observed effect size *d* can be calculated as the number of SDs difference between two improvement scores:

$$d=\frac{\left(i\,m\,p\,r\,o\,v\,e\,m\,e\,n\,t\,\;s\,c\,o\,r\,e\,\;o\,f\,\;g\,r\,o\,u\,p\,\;1-\;i\,m\,p\,r\,o\,v\,e\,m\,e\,n\,t\,\;s\,c\,o\,r\,e\,\;o\,f\,\;g\,r\,o\,u\,p\,\;2\right)}{S\,D}$$

where the SD is the pooled SD^12^. In our case, group 1 is the Bimodal group and group 2 the Unimodal group.

**FIGURE 5:**
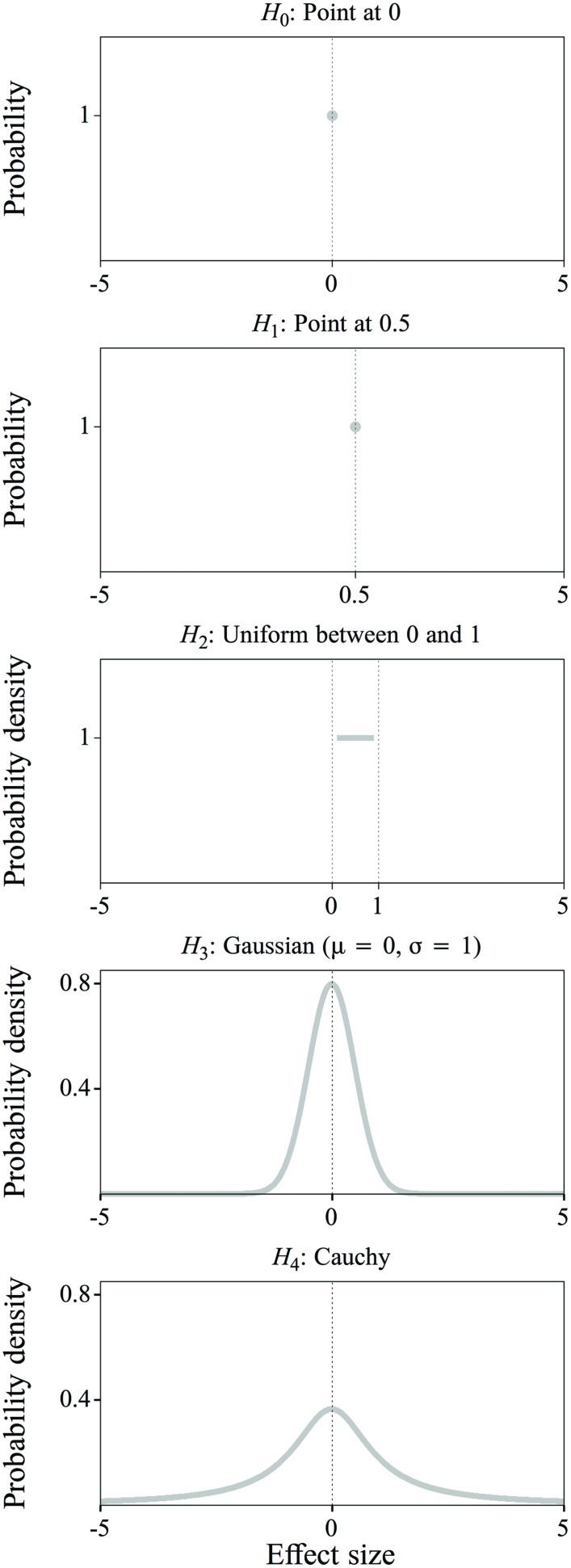
**Null hypothesis (*H_0_*) and four alternative hypotheses (*H_1_* through *H_4_*) about the effect size: a point distribution at 0 (*H_0_*), a point distribution at 0.5 (*H_1_*), a uniform distribution between 0 and 1 (*H_2_*), a Gaussian distribution with mean = 0 and sigma = 1 (*H_3_*) and a Cauchy distribution (*H_4_*).** Explanation: see text.

The null hypothesis (**Figure 5**, top) is always the same, namely that there is no difference in the improvement score between the Unimodal and Bimodal groups, and that accordingly the effect size *d* is exactly zero:

$$H_{0}\,\;:\;d=0$$

The value of the BF depends on the definition of the alternative hypothesis. To accommodate different *a priori* beliefs about the effect size, we computed the BF in four different ways, i.e., with four different alternative hypotheses, which are increasingly less specific about the expected value of the effect size. The first and second alternative hypotheses (*H_1_* and *H_2_*) include information about the effect size obtained from EBW2011, WER2013, and WB2013; the third and fourth alternative hypotheses (*H_3_* and *H_4_*) do not. **Table 4** provides an overview of the four alternative hypotheses and the resultant BFs, which we will now discuss in detail^13^.

**Table 4:** The four alternative hypotheses (H) and the resulting Bayes factors (BF).

H		BF
H_1_:	*d* = +0.50	BF_01_ = 137.86
H_2_:	*d* is a random value drawn from a uniform distribution between 0 and 1.	BF_02_ = 5.97
H_3_:	*d* is a random value drawn from a Gaussian distribution with mean 0 and SD 1.	BF_03_ = 5.32
H_4_:	*d* is a random value drawn from a Cauchy distribution	BF_04_ = 4.73

Alternative hypothesis 1 (**Figure 5**, second from top) stipulates that the effect size *d* is a specific value:

$$H_{1}\,\;:\;d=+0.50$$

This value of +0.50 is based on effect sizes derived from the improvement scores observed in EBW2011, WER2013, and WB2013, as follows. In EBW2011 and WER2013, one group of listeners was exposed to a non-enhanced bimodal distribution (the Bimodal group), a second group to an enhanced bimodal distribution (the Enhanced group), and a third group to music (the Music group). In WB2013, improvement in categorization was compared between a Music group and two Enhanced groups, one presented with a discontinuous distribution and the other with a continuous distribution. As mentioned in the Introduction (see “No Adequate Control for Dispersion Across Distributional Learning Studies”), in all three studies the improvement score was significantly larger for the Enhanced group than for the Music group. In EBW2011 and WER2013, the improvement score for the Bimodal group was not significantly different from that of the Music group and also not from that of the Enhanced group. For the current analysis, we considered the improvement scores of the previous Enhanced groups as proxies for the expected improvement score of our Bimodal group (which was also exposed to an enhanced bimodal distribution, just as the Enhanced groups in the previous studies; see “Solving the Problems: an Equally Wide Unimodal Control Distribution”). Because it was not clear whether our Unimodal group would behave more similarly to the previous Music groups or to the previous Bimodal groups, we considered the improvement scores of the previous Music and Bimodal groups as proxies for the expected improvement score of our Unimodal group. When calculating the effect sizes observed in the three studies, we used the above-mentioned formula for the effect size *d*, and took a previous Enhanced group as group 1, and either a previous Bimodal group or a previous Music group as group 2. The improvement scores for the Enhanced, Bimodal and Music groups were 6.04% (CI = +2.76 ∼ +9.31%), 0.80% (CI = –2.22 ∼ +3.83%) and –0.15% (CI = –3.50 ∼ +3.21%) respectively in EBW2011, and 6.63% (CI = +4.05 ∼ +9.20%), 3.83% (CI = +0.97 ∼ 6.68%) and 2.00% (CI = –0.50 ∼ +4.50%) respectively in WER2013. The improvement scores for the Enhanced and Music groups in WB2013 were 9.68% (CI = +6.80 ∼ +12.55%) and 2.00% (CI = –0.50 ∼ +4.50%) respectively^14^. The pooled SD for the Enhanced and Bimodal groups was 12.00% in EBW2011 and 9.57% in WER2013. The pooled SD for the Enhanced and Music groups was 12.09% in EBW2011, 8.94% in WER2013 and 9.50% in WB2013. **Table 5** shows the resulting effect sizes *d*.

**Table 5:** Effect size *d* in previous studies (see text).

Previous study	Enhanced–Bimodal	Enhanced–Music
EBW (2011)	+0.44	+0.51
WER (2013)	+0.29	+0.52
WB (2013)		+0.81

The average of the five listed effect sizes is +0.51, which we rounded to +0.50 in hypothesis 1. Notice that this value is explicitly positive, i.e., it reflects the belief that our Bimodal group will have a *higher* improvement score, and thus improve *more* after distributional training than the Unimodal group. The BF calculated on the basis of the null hypothesis versus this first alternative hypothesis expresses strong support for the null:

$$B\,F_{01}=137.86$$

Specifically, BF_01_ indicates that the observed data are 137.86 times more likely to have occurred under *H_0_* (that *d* is exactly 0), than under *H_1_* (that *d* is exactly 0.5).

In alternative hypotheses 2 through 4, the effect size is no longer defined as a specific value, but as a probability density function (**Figure 5**, as explained below): *d* is expected not to be one specific value, but a random value drawn from a distribution whose form defines the likelihood of that value. In alternative hypothesis 2, the effect size is any value between 0 and 1 with equal probability (**Figure 5**, middle):

*H*_2_: *d* is a random value drawn from a uniform distribution between 0 and 1.

The hypothesis still includes the information mentioned in **Table 5** about previously obtained effect sizes (i.e., all effect sizes in **Table 5** fall within the range of the distribution), but it is vaguer about the precise value of the expected effect size than hypothesis 1. Since *d* is defined as 0 or positive, hypothesis 2 expresses the belief that the Bimodal group will improve *at least as much* as the Unimodal group. The BF calculated on the basis of the null hypothesis versus this second alternative hypothesis also expresses support for the null:

$$B\,F_{02}=5.97$$

That is, BF_02_ implies that the observed data are 5.97 times more likely to have occurred under *H_0_* (that *d* is exactly 0) than under *H_2_* (that *d* is somewhere between 0 and 1).

Hypotheses 1 and 2 show that previous observations can be incorporated in the alternative hypothesis to different extents, depending on the researcher’s belief in the truth value of these observations. Previous observations can also be deemed inappropriate for incorporation in the alternative hypothesis, for example if concerns (such as mentioned in “Problems in Previous Research on Distributional Learning”) about the earlier observations create uncertainty about the applicability of the information to the experiment to be performed. In this case, the alternative hypothesis should reflect the assumption that we do not have a clear expectation about the effect size. This is done in alternative hypotheses 3 and 4. In alternative hypothesis 3, the effect size is any value around 0, with values closer to the mean being more likely than values further away from the mean as defined by a Gaussian distribution (**Figure 5**, fourth from top):

*H*_3_: *d* is a random value drawn from a Gaussian distribution with a mean of 0 and a SD of 1.

Since *d* can be positive, zero, or negative, the belief that the Bimodal group will improve at least as much as the Unimodal group, which was inherent in alternative hypotheses 1 and 2, is now dropped. The BF calculated on the basis of the null hypothesis versus the third alternative hypothesis still expresses support for the null:

$$B\,F_{03}=5.32$$

In other words, BF_03_ indicates that the observed data are 5.32 times more likely to have occurred under *H_0_* (that *d* is exactly 0) than under *H_3_*, (that *d* is a value around zero, whose probability is defined by a Gaussian distribution).

It is possible to be even less specific about the expected value of the effect size than in alternative hypothesis 3, by loosening the belief that the effect size is more likely to occur close to zero. This is done with a Cauchy distribution (for an explanation, see Rouder et al., 2009), as used in alternative hypothesis 4 (**Figure 5**, bottom):

*H*_4_: *d* is a random value drawn from a Cauchy distribution, with a width of (√2)/2.^15^

Notice in **Figure 5** that the tails of the Cauchy distribution are much heavier than those of the Gaussian distribution, thus reflecting a much smaller confidence that the effect size should be relatively close to zero. Again, the BF calculated on the basis of the null hypothesis versus the fourth alternative hypothesis expresses support for the null:

$$B\,F_{04}=4.73$$

Thus, BF_04_ indicates that the observed data are 4.73 times more likely to have occurred under *H_0_* (that *d* is exactly 0) than under *H_4_* (that *d* is a value around zero, whose probability is defined by a Cauchy distribution, i.e., with more uncertainty as to the effect size than expressed in the Gaussian distribution used for *H_3_*).

In sum, four different calculations of the Bayes factor, which differ in the extent to which they incorporate *a priori* beliefs about the expected effect size, unanimously support the null hypothesis that there is no difference between bimodally and unimodally trained Spanish participants in improvement of categorization of Dutch [ɑ]- and [a]-tokens. If we follow the interpretation of Bayes factors by Kass and Raftery (1995; see above in this section), the support for the null hypothesis ranges from moderate support (hypotheses 2 through 4, which represent less strong *a priori* beliefs about the effect size than hypothesis 1) to strong support (hypothesis 1, which incorporates the most explicit *a priori* beliefs).

## Discussion

In the present study we trained Spanish adult participants on a bimodal or a unimodal distribution encompassing the Dutch vowel contrast /ɑ/∼/a/, and then tested their improvement in categorization of Dutch [ɑ]- and [a]-tokens after training. For the first time in the research on distributional learning of speech sounds, the bimodal and unimodal distributions had nearly identical dispersions, as defined by the range, SD and edge strength. The results show that Spanish adult participants improve their categorization of Dutch [ɑ]- and [a]-tokens irrespective of the training distribution, and that categorization accuracy does not improve significantly more after exposure to one distribution than after exposure to the other distribution. Additionally, four different Bayes factors (ranging from incorporating *a priori* beliefs about the expected effect size as much as possible to not incorporating previous knowledge at all) provided unanimous evidence for the null hypothesis that there is no difference between bimodally and unimodally trained Spanish listeners in categorization improvement. In other words, the number of peaks in the distribution does not play a role in the observed improved categorization.

The number of peaks must now also be dismissed as the factor that explains the earlier results on Spanish listeners’ larger improved categorization of Dutch [ɑ]- and [a]-tokens after enhanced bimodal training than after listening to music (Escudero et al., 2011; Wanrooij and Boersma, 2013; Wanrooij et al., 2013; Escudero and Williams, 2014). Future research should determine which factor(s) do account for these results. At least two factors, which were also mentioned in the Introduction, appear to be viable candidates: “processing speech versus non-speech” (since the earlier studies compared learning from exposure to a speech distribution to learning from exposure to non-speech) and the “wide dispersion” of the enhanced bimodal distributions (since the earlier studies compared learning from exposure to an enhanced bimodal distribution to learning from exposure to music, which has no relevant dispersion).

The conclusion that the number of peaks in the distributions cannot explain the observed perceptual learning in Spanish adults may very well extend to *all* previous results on distributional learning in infants and adults. Although other studies included a control group exposed to a unimodal speech distribution (so that “processing speech versus non-speech” cannot be a factor accounting for the reported effects), none of the studies controlled for dispersion as was done in the current study. Results from other paradigms than distributional training suggest that enhancement of training stimuli (i.e., a wide dispersion in the training distributions) can advance the learning of speech sound categories through drawing participants’ attention to the relevant differences between the categories (e.g., Jamieson and Morosan, 1986; Iverson et al., 2005; Kondaurova and Francis, 2010). In view of this potential influence of dispersion on attentional learning, dispersion is a high-ranking potential confounding factor whose role should be separated from that of the number of peaks before we can conclude that distributional learning based on the number of peaks is a mechanism that tunes speech perception.

## Conflict of Interest Statement

The authors declare that the research was conducted in the absence of any commercial or financial relationships that could be construed as a potential conflict of interest.

## Footnotes

1 The true bimodal means are somewhat closer together than the two peaks.

2 Specifically, the values in Escudero et al. (2011) and Wanrooij et al. (2013) were as follows. In the non-enhanced bimodal distribution, the distance between the peaks was 0.67 ERB, the range was 12.60–13.54 ERB, and the SD of the pooled distribution was 0.31 ERB. In the enhanced bimodal distribution, the distance between the peaks was 2.02 ERB, the range was 11.52–14.35 ERB, and the SD was 0.93 ERB.

3 Note that enhancement of the contrast reduces the overlap between the categories if the SDs of each peak remain the same. The overlap is not necessarily reduced if the SD of each peak is increased as well (as it is in **Figure 2**).

4 In Escudero and Williams (2014), who investigated longer-term effects of distributional training (i.e., after 6 and 12 months rather than only after a few minutes), a significant difference between listeners to an enhanced bimodal distribution and listeners to music was only found in a subset of the tests.

5 Notice that the SDs of the *distributions* are compared, not those of the *individual peaks*. (In **Figure 3**, the SDs of the individual peaks would be 0.8 for each peak in the bimodal distribution and 1.7 for the unimodal peak). A smaller SD of each bimodal peak than of the unimodal peak is not problematic in a distributional-learning experiment, because it supports the experimental design. Specifically, in the bimodal distribution both the presence of two peaks and the smaller SD of each peak than in the unimodal distribution promote the distributional learning of two separate categories, while conversely in the unimodal distribution both the presence of a single peak and the larger SD of this peak than in the bimodal distribution promote distributional learning of a single category (Guenther and Gjaja, 1996).

6 The formula used for the skewed bimodal distribution is: exp (-0.5 ^∗^((*x* - μ1)/σ) ˆ 2) + exp (-0.5 ^∗^ ((*x* - μ2)/σ) ˆ 2) + 0.2 ^∗^ exp (-0.5 ^∗^ ((*x* - 0.50)/σSkew) ˆ 2), where μ1 and μ2 are 20 and 80% of the range respectively, σ is 10% of the range, and σSkew is set at 15% of the range. (The first two elements are the sum of the two Gaussian curves, the last element adds the skew).

7 Notice that the SDs of the Gaussians defining the shape of the distributions (e.g., 100% of the range for the unimodal distribution) are not identical to the SDs of the peaks in the distributions used in the experiment (e.g., 28.4% of the range for the unimodal distribution), which are not truly Gaussian. This is because the tails of the unimodal and bimodal distributions are cut off at the maximum and minimum acoustic values of F1 and F2, and because the bimodal distribution is a *sum* of two Gaussians.

8 The F1 and F2 values of the two response options in the test in EBW2011, WER2013, and WB2013 were for [ɑ] F1 = 12.5 ERB, F2 = 16.1 ERB and for [a] F1 = 13.3 ERB, F2 = 17.4 ERB.

9 In the region of Dutch /i/ and /y/ in the F1–F2 vowel space, Spanish has the vowel /i/ only. However, Spanish listeners tend to hear a rather clear difference between tokens of Dutch /i/ and /y/, possibly because the rounding of /y/ makes them perceive tokens of /y/ as close to Spanish /u/ (Escudero and Wanrooij, 2010). Listeners in the current experiment, as in EBW2011, WER2013, and WB2013, did not show any difficulties with the practice test.

10 The effect sizes *d* are calculated as: (the group’s mean improvement)/(the SD of the improvements of the group members).

11 The calculation of effect size *d* is explained in “Bayes Factors.”

12 The pooled SD is calculated as the within-sums-of-squares/(N1 + N2 - 2).

13 The four Bayes factors can be computed in R (R Core Team, 2013) with the equation **dt** (*t*, *df*)/(**mean** (*weight* ^∗^**dt** (*t*, *df*, **ncp** = *d* ^∗^**sqrt**(*n*)))/**mean** (*weight*)). In this equation, **dt** is the R function that computes the *t* probability density, and **ncp** is the non-centrality parameter of this density; *t* is the between-groups *t* value of our experiment, i.e., -0.43; *df* is the number of degrees of freedom for a *t*-test, i.e., 60 + 60 - 2 = 118; *n* is half the geometric mean of the two group sizes (Rouder et al., 2009, p. 234), i.e., 60^∗^60/(60 + 60) = 30; *d* is the hypothesized range of possible effect sizes, and *weight* is the shape of the distribution for all these *d* values. For *H*_1_, *d* is 0.5 and *weight* is 1. For *H*_2_, *d* is (-0.5+1:1e5)/1e5 and *weight* is 1. For *H*_3_, *d* is ((-10e5^∗^*width* + 0.5):(10e5^∗^*width*-0.5))/1e5 and *weight* is exp(-0.5^∗^(*d*/*width*)ˆ2), where *width* is 1. For *H*_4_, *d* is ((-1000^∗^1e4^∗^*width* + 0.5):(1000^∗^1e4^∗^*width*-0.5))/1e4 and *weight* is 1/(1 + (*d*/*width*)ˆ2)), where *width* is sqrt(2)/2 (our equations for *H*_3_ and *H*_4_ are formulated in such a way that they will also work for other values of *width*). At the time of writing the computations for *H*_3_ and *H*_4_ are also available on Rouder’s website (http://pcl.missouri.edu/bayesfactor).

14 The Enhanced group referred to here is the group presented with a continuous enhanced distribution in WB2013 (the Continuous Enhanced group). In WB2013 the group presented with a discontinuous enhanced distribution (the Discontinuous Enhanced group) and the Music group were taken from WER2013.

15 The equation used for the Cauchy distribution in R is: ((-1000^∗^1e4^∗^*width* + 0.5):(1000^∗^1e4^∗^*width*-0.5))/1e4, where *width* is sqrt(2)/2 (see also note 13).
